# Cholesterol and Alzheimer’s Disease; From Risk Genes to Pathological Effects

**DOI:** 10.3389/fnagi.2021.690372

**Published:** 2021-06-24

**Authors:** Femke M. Feringa, Rik van der Kant

**Affiliations:** ^1^Department of Clinical Genetics, Center for Neurogenomics and Cognitive Research (CNCR), Amsterdam University Medical Center, Amsterdam, Netherlands; ^2^Department of Functional Genomics, Center for Neurogenomics and Cognitive Research (CNCR), VU University Amsterdam, Amsterdam, Netherlands; ^3^Alzheimer Center Amsterdam, Department of Neurology, Amsterdam Neuroscience, Amsterdam University Medical Center, Amsterdam, Netherlands

**Keywords:** cholesterol, Alzheimer, ApoE, gliosis, iPSC models

## Abstract

While the central nervous system compromises 2% of our body weight, it harbors up to 25% of the body’s cholesterol. Cholesterol levels in the brain are tightly regulated for physiological brain function, but mounting evidence indicates that excessive cholesterol accumulates in Alzheimer’s disease (AD), where it may drive AD-associated pathological changes. This seems especially relevant for late-onset AD, as several of the major genetic risk factors are functionally associated with cholesterol metabolism. In this review we discuss the different systems that maintain brain cholesterol metabolism in the healthy brain, and how dysregulation of these processes can lead, or contribute to, Alzheimer’s disease. We will also discuss how AD-risk genes might impact cholesterol metabolism and downstream AD pathology. Finally, we will address the major outstanding questions in the field and how recent technical advances in CRISPR/Cas9-gene editing and induced pluripotent stem cell (iPSC)-technology can aid to study these problems.

## Introduction

Dementia affects over 46 million people worldwide, a number that is expected to double within the next 20 years due to our increased life expectancy (Prince et al., 2015). Alzheimer’s Disease (AD) is the most common type of dementia and no successful treatment that can cure AD, or halt its progression, is available today. At the pathological level, AD is characterized by the accumulation of extracellular amyloid beta (Aβ) plaques, and intracellular neurofibrillary tangles (NFT) consisting of hyperphosphorylated Tau species (Scheltens et al., 2016). AD can develop early (<65 years) referred to as early-onset AD (EOAD), which is in part explained by autosomal dominant inheritance of coding mutations in the amyloid precursor protein (APP) or presenilin genes (*PSEN1* and *PSEN2*), in that case called familial AD (FAD). The FAD related mutations directly affect Aβ production and their identification therefore contributed to formation of the amyloid cascade hypothesis that postulates a linear relation between development of Aβ plaques and NFT in AD (Pimenova et al., 2018). Whilst EOAD only represents around 5% of all AD cases, the vast majority of AD patients suffer from late-onset AD (LOAD), for which aging is the biggest risk factor in addition to genetic and lifestyle factors (Scheltens et al., 2016). Multiple studies on the lifestyle and genetic interactions with AD have connected altered circulating cholesterol metabolism and hypercholesterolemia with aging and AD pathogenesis (Box 1). In the brain, already a century ago, in addition to plaques and tangles, Dr. Alzheimer described as a third characteristic of AD: the extensive accumulation of ‘adipose saccules’ (Alzheimer, 1907). These ‘adipose saccules’ were likely what we now refer to as lipid droplets, and are major storage organelles of intracellular lipids such as cholesterol and fatty acids (Farmer et al., 2020). While mostly ignored since their first discovery, these ‘adipose saccules’ in the brain have gained renewed interest in light of the findings on cholesterol metabolism and AD in the last two decades.

**Box 1. Hypercholesterolemia, high fat diet and AD.**

With the identification of lipoprotein ApoE4 as the biggest risk factor for LOAD in the early nineties, the interest for lipids and cholesterol metabolism in AD rapidly developed (Corder et al., 1993; Saunders et al., 1993; Strittmatter et al., 1993). Expression of the ApoE4 allelic variant of ApoE had previously been shown to increase plasma low-density lipoprotein (LDL) levels and increase the risk for atherosclerosis. Moreover, carriers of the ApoE4 gene were overrepresented in hyperlipidemic and cardiovascular patients (Huang, 2010; Mahley, 2016). At the epidemiological level, retrospective studies have shown that obesity, type 2 diabetes, cardiovascular disease and hypercholesterolemia at middle-age increase the risk for dementia at older age in humans (Pappolla et al., 2003; Whitmer et al., 2005; Stampfer, 2006; Solomon et al., 2009; Pedditizi et al., 2016; Anstey et al., 2017; Ma et al., 2020; Tini et al., 2020; Barbiellini Amidei et al., 2021). In line, increased plasma and CSF levels of the cholesterol metabolite 24-hydroxycholesterol (24S-OHC) that is selectively generated in neurons, have been linked to early AD development (Lütjohann et al., 2000; Papassotiropoulos et al., 2002; Schönknecht et al., 2002; Li et al., 2018). Cholesterol has also been shown to accumulate in mature Aβ-plaques in AD patients and APP(SW) mice (Mori et al., 2001) and cholesterol levels in the brain positively correlate with the severity of dementia in AD patients (Cutler et al., 2004). In line, lower AD incidence was associated with statin use in retrospective studies (Jick et al., 2000; Wolozin et al., 2000; Cramer et al., 2008; Haag et al., 2009; Li et al., 2010; Lin et al., 2015; Zissimopoulos et al., 2017; Zhang et al., 2018). The protective effect of statin usage was present independent of ApoE status (Haag et al., 2009; Li et al., 2010). A subset of these studies showed that the protective effect of statin usage was no longer present in participants that fell within the oldest age categories (>80). This could be due to a survival bias, where participants that survive till old age have fewer additional medical conditions, that would have increased their risk for AD development. Alternatively, this could point to a beneficial effect of statin usage only when taken at a timepoint before pathological hallmarks of AD would typically develop in the brain. A link between high circulating cholesterol levels and AD was also corroborated in AD mouse models where a hypercholesterolemic diet increased Aβ-plaque load (Refolo et al., 2000). In addition, high cholesterol diet in mice induced Tau hyperphosphorylation, which was amplified by loss of ApoE expression (Rahman et al., 2005; Glöckner et al., 2011). Glial activation, contributing to gliosis as seen in AD, has also been reported in mice on a high cholesterol diet (Crisby et al., 2004). Metabolic changes accompanied by AD phenotypes in the brain, where also described in rabbits on a high cholesterol diet (Jin et al., 2018). Finally, a high fat/high cholesterol diet in young (4-month old) versus aged (14-month old) rats negatively affected memory performance at both ages, while also increasing hippocampal pTau levels at old age, indicating the detrimental combination of disturbed circulating cholesterol homeostasis and aging (Ledreux et al., 2016).

In this review, we will discuss the basic regulation of cholesterol homeostasis at the cellular level, and how crosstalk between different brain-cell types regulates “healthy” cholesterol homeostasis in the human brain. We will then discuss how brain cholesterol metabolism is affected by aging and how neuronal cholesterol can contribute to downstream AD pathologies such as Amyloid- and Tau accumulation. Next, we will examine the contribution of human-specific AD risk polymorphisms to cholesterol dyshomeostasis and gliosis in different brain cell types, an area of research that greatly benefits from the development of CRISPR gene-editing and iPSC techniques. Lastly, we will formulate some of the major questions still outstanding in the field, and how they could be addressed to develop disease-modifying interventions for AD based on our knowledge of cholesterol metabolism in AD.

## Intracellular Cholesterol Metabolism; The Basics

**FIGURE 1:**
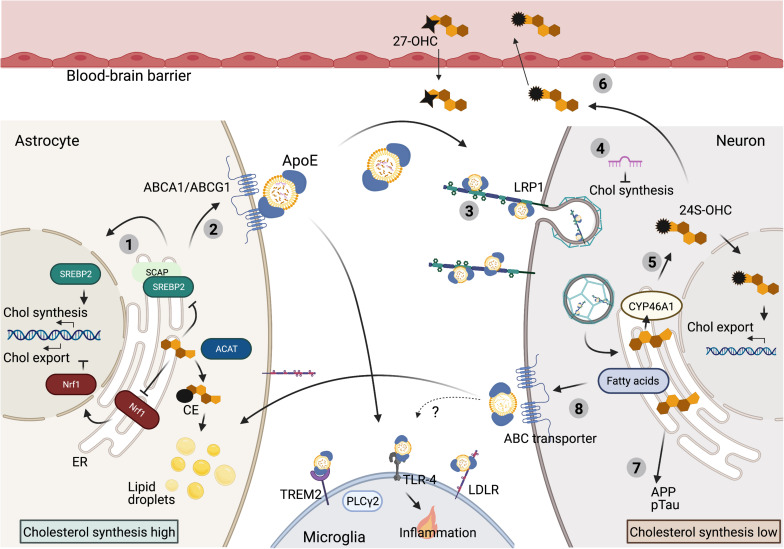
Cholesterol metabolism in the brain. Overview of cholesterol metabolism in the mature brain. (1) In the brain cholesterol is predominantly synthesized in astrocytes. Cholesterol synthesis is under tight control of ER-cholesterol level. High ER-cholesterol concentrations prevent SREBP2 processing and thereby suppress cholesterol synthesis. High ER-cholesterol levels also inhibit Nrf1 processing to induce cholesterol export. In addition, excess ER cholesterol is converted to CE for storage in lipid droplets. (2) Cholesterol and cholesterol precursors are exported via ABC transporters and transported from astrocytes to neurons and microglia via ApoE bound lipoprotein-particles. (3) These lipoprotein particles can bind to lipoprotein receptors (LRP1 on neurons, TREM2, TLR-4 and LDLR on microglia) to be internalized. (4) Neuronal cholesterol synthesis is inhibited by ApoE dependent delivery of astrocytic derived microRNAs that target cholesterol synthesis genes in neurons. (5) Specifically in neurons, excess cholesterol is converted to 24S-OHC, which activates a transcriptional program to promote cholesterol export. (6) 24S-OHC itself can be secreted from the brain via crossing of the BBB, while 27OHC can enter the brain from the periphery. (7) High cholesterol load in neurons can contribute to amyloidogenic APP processing and pTau accumulation. (8) Astrocytes can also prevent toxic overload of (peroxidized) fatty acids in neurons via ApoE-dependent lipid-particle transport from neurons to astrocytes, but whether cholesterol is also transported into this direction remains unknown (BioRender, 2021).

The largely hydrophobic molecule cholesterol localizes primarily in cell membranes where it regulates membrane fluidity and can interact with neighboring lipids and proteins to regulate membrane trafficking or signal transduction (Luo et al., 2019). Cholesterol levels in cells are balanced by *de novo* biosynthesis, uptake, storage and export [extensively reviewed by Luo et al. (2019)]. In short: *De novo* synthesis of cholesterol starts when sterol regulatory element binding protein 2 (SREBP2), the key regulator of cholesterol synthesis, transfers from the endoplasmic reticulum (ER)-membrane to the Golgi where it is processed in order to enter the nucleus and induce transcription of its numerous target genes involved in cholesterol synthesis (Figure 1). Together around 30 consecutive reactions ensure cholesterol synthesis in the ER starting from acetyl-CoA. HMG-CoA reductase (HMGCR) and squalene monooxygenase (SM), both SREBP2 targets, are rate limiting enzymes in this process. Cholesterol is transported from the ER to the plasma membrane without passing through the Golgi (Dai et al., 2021). As an alternative to synthesis, cells can acquire cholesterol trough uptake. When not incorporated in the lipid bilayer of a membrane, most cholesterol is protein bound in apolipoprotein particles that facilitate transport (Zhang and Liu, 2015). Uptake of these cholesterol containing particles depends on Low-density lipoprotein receptors (LDLRs) on the plasma membrane. Binding of lipoprotein particles to the LDLR causes incorporation of LDL into clathrin-coated vesicles that enter the endocytic pathway (Goldstein and Brown, 2009). LDLR is either recycled via endosomal recycling or directed to lysosomes for degradation (Rudenko et al., 2002; Bartuzi et al., 2016; Fedoseienko et al., 2018). When LDLs arrive in lysosomes, cholesterol is freed by hydrolysis of CEs present in the LDLs. NPC2, NPC1 and lysosome-associated glycoprotein LAMP2 control delivery of the newly formed cholesterol to the ER (Kwon et al., 2009). Excess cholesterol can be stored in lipid droplets as CEs and converted back to cholesterol by acidic lipases in the lysosome when needed (Ikonen, 2008). As an alternative to intracellular storage, excess cholesterol can also be exported as part of LDL- or High-density lipoprotein (HDL) particles in a process named reverse cholesterol transport. This is mediated through ATP-binding cassette (ABC) transporters, like ABC subfamily A member 1 (ABCA1) and ABC subfamily G member 1 (ABCG1), which are widely expressed in the body and coordinately regulate cholesterol export from the cell (Figure 1) (Luo et al., 2019). Although the exact mechanism is still under debate, cholesterol effluxed by ABCA1 appears to be loaded on lipid-free Apolipoprotein A-I (ApoA-I), which can subsequently acquire additional cholesterol from ABCG1 (Gelissen et al., 2006).

While representing only 1% of cellular cholesterol, cholesterol levels in the ER play a central role in the regulation of all aspects of cholesterol metabolism described above. When cholesterol levels are low, SREBP2 interacts with SCAP in the ER membrane which promotes SREBP2 trafficking to the Golgi, it’s processing and transcription of cholesterol-synthetic genes (Sakai et al., 1997, 1998; Brown et al., 2018). The uptake of cholesterol is also directly regulated through this process, as LDLR is a transcriptional target of SREBP2 (Luo et al., 2019). In this way, low ER cholesterol drives increased synthesis and uptake of cholesterol in order to balance cellular cholesterol levels (Figure 1). Reversely, too high levels of cholesterol can be toxic to cells. Therefore, when a surplus of cholesterol accumulates in the plasma membrane, cholesterol is transported back to the ER where it (i) inhibits SREBP2 activation and (ii) can be esterified by acyl-coA: cholesterol acyltransferase (ACAT1) to from CE for storage in lipid droplets (Chang et al., 1997; Zhang and Liu, 2017). Furthermore, when cholesterol levels in the ER are high, cholesterol is converted to oxysterols (Olsen et al., 2012). As the major sensor for cholesterol overload in a cell oxysterols prevent SREBP2 activation and directly activate the Liver × receptor (LXR), which promotes cholesterol efflux by transcription of ABC transporters (Radhakrishnan et al., 2007; Olsen et al., 2012). In addition to SREBP2, the transcription factor Nuclear factor erythroid 2-related factor 1 (NFE2L1 aka Nrf1) also senses ER cholesterol levels. When ER cholesterol levels are low, Nrf1 is cleaved and the transcription-part domain enters the nucleus where it inhibits LXR dependent transcription and thus prevents cholesterol export. When ER cholesterol levels rise, cholesterol binding to Nrf1 prevents its translocation to the nucleus, which causes de-repression of the LXR locus and promotes cholesterol efflux (Figure 1) (Widenmaier et al., 2017). Together, in a Yin-Yang manner, SREBP2 and Nrf1 sense ER cholesterol levels to maintain cellular cholesterol homeostasis (Figure 1).

It is important to note, that many basic rules underlying intracellular cholesterol metabolism have been uncovered so far, as discussed above. However, most of this knowledge is acquired from experiments in dividing fibroblast culture systems. Cholesterol metabolism in the central nervous system (CNS) and, particularly in neurons, is under added pressure due to the postmitotic nature of these cells, their long-life span, large size and highly specialized metabolic demand which requests specific mechanisms to maintain lifelong cholesterol homeostasis in the brain.

## Cholesterol Metabolism in the Brain; Different Needs for Different Cells

Due to the blood-brain barrier (BBB) cholesterol metabolism in the CNS is largely separated from the periphery and it is generally understood that little diet-derived cholesterol enters the brain (Björkhem and Meaney, 2004). Therefore the brain is largely dependent on its own cholesterol synthesis and separate metabolism regulated by a complex interplay between different highly specialized cell types, each with their own demand for cholesterol (Dietschy and Turley, 2004). In adults, biosynthesis of cholesterol is thought to almost exclusively take place in astrocytes, from where cholesterol is transported to neurons via ApoE lipoproteins (Figure 1) (Pfrieger and Ungerer, 2011). Although cell type specific cholesterol synthesis rates have not been determined *in vivo*, cholesterol synthesis rates in cultured rat astrocytes are double as high as in cultured neurons (Nieweg et al., 2009). Moreover, conditional depletion of cholesterol synthesis in neuronal cells in mice did not result in neurodegeneration or synapse loss, indicating that mature neurons can acquire sufficient cholesterol levels supplied by surrounding glia (Fünfschilling et al., 2007). Accordingly, neuronal synaptogenesis has been shown to depend on ApoE dependent cholesterol transport from astrocytes to neurons (Mauch et al., 2001; Pfrieger, 2003). Of interest is the recent finding that astrocytes might also suppress cholesterol synthesis in neurons, as astrocytic ApoE was shown to deliver microRNAs to neurons that target and suppress expression of cholesterol biosynthesis genes (Li et al., 2021). Astrocyte-derived lipoproteins carry cholesterol and phospholipids as well as cholesterol precursors, presumably used by neurons for processing, but contain little CE or triglycerides making them substantially different from plasma lipoproteins (Pfrieger and Ungerer, 2011). Instead of ApoA-I, ApoE is the main apolipoprotein responsible for lipid transport in the CNS. ApoE is highly expressed in astrocytes where it is lipidated and exported via ABC transporters like ABCA1 and ABCG1 (Figure 1) (Koldamova et al., 2003; Xu et al., 2006). Which ABC transporters are responsible for cholesterol efflux in the CNS seems to be cell-type dependent. Blocking ABCA1 or ABCG1 mediated transport in primary rat astrocytes reduced ApoE mediated cholesterol export, but had no effect on cholesterol efflux from primary cultured neurons. In contrast, knock down of ABCG4 selectively affected cholesterol export in primary cultured neurons (Chen et al., 2013). Neurons can take up astrocyte-derived HDL-like lipoprotein particles containing ApoE through receptors of the LDLR family (Figure 1), of which LRP1 is highest expressed in neurons (Vance and Hayashi, 2010). Similar to neurons, cholesterol biosynthesis levels are relatively low in microglia, which also mainly depend on astrocytes for cholesterol production (Zhang et al., 2014; Loving and Bruce, 2020). On the microglial cell surface, ApoE lipoprotein particles can interact with Triggering Receptor Expressed on Myeloid Cells 2 (TREM2), Toll Like Receptor 4 (TLR-4) and the LDLR to internalize lipoprotein particles into the microglia (Figure 1) (Loving and Bruce, 2020).

As mentioned above, regulation of cholesterol metabolism is particularly important for neurons. To further fine-tune cholesterol metabolism, neurons contain another cholesterol-regulating enzyme; cholesterol 24-hydroxylase (CYP46A1), which is CNS specific and under healthy conditions only expressed by neurons (Brown et al., 2004; Ramirez et al., 2008). CYP46A1 converts excess cholesterol to 24S-hydroxycholesterol (24S-OHC) (Lund et al., 2003; Ramirez et al., 2008; Zhang and Liu, 2015; van der Kant et al., 2019), which can be released by neurons and crosses the BBB through diffusion, forming a major export pathway for excess cholesterol from the brain (Figure 1) (Lütjohann et al., 1996; Lund et al., 1999, 2003; Xie et al., 2003). Due to its neuron specific origin, 24S-OHC levels in the blood also provide a direct measure of cholesterol turnover levels in the brain (Sodero, 2020). Besides being an export product, as other oxysterols, 24S-OHC can promote ApoE-mediated cholesterol export by activating liver X receptor (LXR) (Figure 1) (Abildayeva et al., 2006; Matsuda et al., 2013). Additional oxysterols that are produced in the brain include 27-OHC, which is generated by the enzyme CYP27A1 and can be further processed by CYP7B to form 7α-hydroxy-3-oxo-4-cholestenoic acid (7-OH-4-C). 7-OH-4-C can cross the BBB to be eliminated by the liver. CYP27A1 is expressed in multiple brain cell types, yet 27-OHC levels in the brain are only a fraction of the far more abundant 24S-OHC (Brown et al., 2004; Heverin et al., 2004; Gilardi et al., 2009). In fact most 27-OHC is not produced in the brain but enters the brain via the BBB originating outside the CNS (Gamba et al., 2015).

While neurons depend on the astrocyte-to-neuron lipid shuttle for supply of cholesterol and cholesterol precursors, recent studies have shown that lipids under certain circumstances can also be transported from neurons to astrocytes. For example, neuronal lipids can become peroxidized when they encounter oxidative stress, potentially generated due to hyperactivity or as an incidental of aging. Neurons are not well equipped to deal with these toxic lipids, and peroxidized lipids in neurons are therefore transported to astrocytes in an ApoE-dependent manner (Figure 1) (Liu et al., 2015, 2017; Moulton et al., 2021; Qi et al., 2021; Ioannou et al., 2019). Astrocytes store these lipids while also upregulating expression of genes responsible for oxidative energy metabolism to process these peroxidized lipids, thereby protecting neuronal integrity (Ioannou et al., 2019). While this directional of lipid-transport is now well established for fatty acids, it is unknown how this neuron-to-astrocyte lipid shuttle affects cholesterol transport and overall cholesterol metabolism in the healthy brain.

## Brain Cholesterol Metabolism; Changes From Development to Aging

The processes that maintain cholesterol homeostasis in the healthy brain are not static, but change during early human development and later again in the aging brain. Cholesterol synthesis rates are at the highest-level during brain development to support the generation of an extensive neuronal network (Waelsch et al., 1940; Quan et al., 2003; Pfrieger and Ungerer, 2011). Therefore, all brain cell types, including both neurons and glial cells are thought to contribute to cholesterol biosynthesis during development (Pfrieger and Ungerer, 2011; Genaro-Mattos et al., 2019). Once an adult, brain cholesterol synthesis rate declines and astrocytic cholesterol production ensures sufficient levels to support neuronal plasticity and glial performance (Andersson et al., 1990; Lütjohann et al., 1996; Dietschy and Turley, 2004; Zhang and Liu, 2015). A further decline in cholesterol synthesis upon aging is suggested by detection of lower cholesterol precursor levels in post mortem hippocampal tissue from middle-aged and elderly (>38 years) donors compared to young (<38 years) donors (Thelen et al., 2006). Yet, absolute cholesterol levels were stable in aged human hippocampal tissue, while a decrease is observed in white and gray matter regions (Söderberg et al., 1990; Thelen et al., 2006). Hippocampal 24S-OHC levels showed a downward trend in middle-aged and elderly (>38 years) donors, which also suggests a decrease in cholesterol metabolism and cholesterol turnover (Thelen et al., 2006). Possibly, lower cholesterol turnover helps to keep cholesterol levels relatively stable in the aging brain when cholesterol synthesis decreases, but less cholesterol turnover might also contribute to reduced neuronal plasticity associated with aging (Thelen et al., 2006). In addition, the BBB, which normally separates CNS and peripheral cholesterol, has been shown to lose integrity during aging (Montagne et al., 2015). This might affect brain cholesterol levels especially in the hippocampus where BBB break-down has been reported to occur first (Montagne et al., 2015; Segarra et al., 2021). Indeed in mice, BBB breakdown results in entry of peripheral cholesterol into the brain, and reversely BBB breakdown also led to increased release of 24S-OHC from the brain into the circulation (Saeed et al., 2014). Brain cholesterol synthesis was increased upon BBB disruption in mice, which might be induced to compensate for the lowered 24S-OHC level (Saeed et al., 2014). What happens to intracellular levels of cholesterol in neurons, astrocytes and microglia during the aging process or downstream of BBB breakdown is not well known.

Age-associated neurodegeneration itself also has a major impact on brain cholesterol metabolism. For example, dying neurons generate high levels of cholesterol-rich debris, in part due to the dismantling of myelin sheets formed by oligodendrocytes. This debris is subsequently phagocytosed by microglia (Callaghan et al., 2014). Recently, Cantuti-Castelvetri et al. (2018) showed that in the aged brain, phagocytes (mainly representing microglia) had lost the ability to process excess amounts of cholesterol, which depended on ApoE and led to accumulation of cholesterol into crystals in the phagocytic cells. The intracellular accumulation of cholesterol in microglia induced an inflammatory response and prevented successful re-myelination. Re-myelination could be restored by stimulation of reverse cholesterol transport or inhibition of the inflammatory response, indicating that aging can affect the cholesterol efflux capacity of immune cells in the brain, which perturbs timely reversal of immune responses needed for proper re-myelination (Cantuti-Castelvetri et al., 2018). Increased presence of lipid droplets, which are storage sites for neutral lipids like glycerolipids and CEs has also been observed in aged microglia of both mouse and human brains (Shimabukuro et al., 2016; Farmer et al., 2020; Marschallinger et al., 2020). While the lipid composition in these droplets has not been well characterized in humans, lipidomic analysis was performed on lipid-droplet containing microglia from aged mouse hippocampus. These lipid droplets contained predominantly glycerolipids, like triacylglycerols (TAGs), diacylglycerols (DAGs), and monoacylglycerols (MAGs), but little CE, indicating that cholesterol is not a major contributor to this age-related phenotype at least in mice (Marschallinger et al., 2020). Therefore, these so-called Lipid-droplet-accumulating microglia (LDAM) seem to be distinct from the microglia in aged mice that accumulated cholesterol after de-myelination (Cantuti-Castelvetri et al., 2018; Marschallinger et al., 2020). In contrast to the general aging process, in AD intracellular cholesterol accumulation has been broadly reported for a number of cell types, as discussed below.

## Cholesterol and AD, a Dual Driver of Aβ and Tau Pathology in Neurons

### Cholesterol, APP Processing and Aβ Generation in Neurons

**FIGURE 2:**
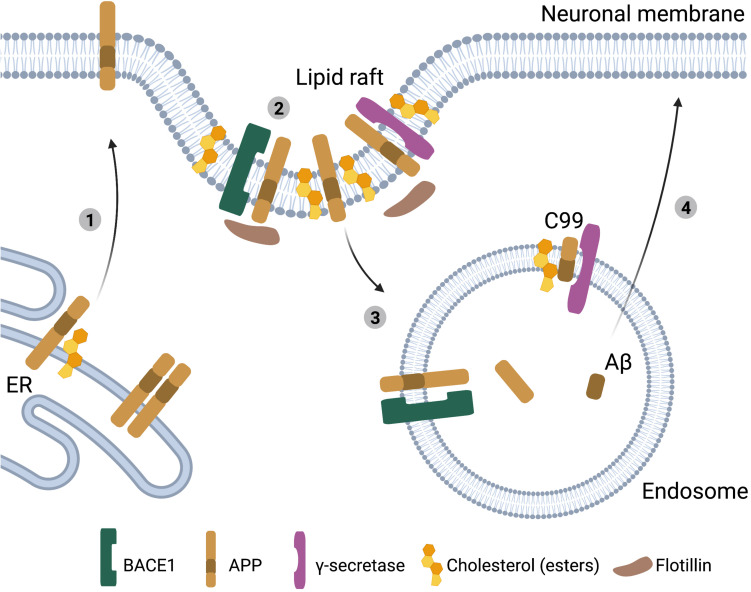
Cholesterol, APP processing and Aβ generation in neurons. Schematic representation of the interactions between cholesterol and APP processing in neurons. (1) High ER-cholesterol levels prevent APP dimerization and promote APP transport to the plasma membrane. (2) Cholesterol or cholesterol esters in the plasma membrane stimulate the clustering of APP, BACE1 and γ-secretases in lipid rafts. (3) These lipid rafts undergo clathrin-mediated endocytosis resulting in amyloidogenic processing of APP to Aβ. (4) Aβ peptides are subsequently secreted from the neuron (BioRender, 2021).

The relationship between cholesterol metabolism, APP processing and Aβ production has been characterized in much detail. Aβ is generated when the amyloid precursor protein (APP) is sequentially processed by β-secretase (BACE1) and γ-secretase. Alternatively, APP can be cleaved by α-secretases, a pathway known as the non-amyloidogenic pathway. APP is normally present in the bilayer membrane of the cell and concentrated in neuronal synapses (Zheng and Koo, 2011). Exogenous addition of cholesterol to human brain tissue lysates promoted BACE1 and γ-secretase activity (Xiong et al., 2008) and treatment of primary mouse neuronal cultures with excess cholesterol was sufficient to increase Aβ_42_ secretion (Marquer et al., 2014). In addition, exogenous cholesterol addition in APP transfected HEK293 cells reduced APP processing via the non-amyloidogenic pathway by α-secretase (Bodovitz and Klein, 1996). Accordingly, lower cholesterol levels have been shown to inhibit APP processing by BACE1 and γ-secretase, while promoting processing of APP via the non-amyloidogenic pathway (Simons et al., 1998; Kojro et al., 2001; Refolo et al., 2001; Schneider et al., 2006; Grimm et al., 2008). These effects of cholesterol on APP processing could be mediated by effects of cholesterol on membrane composition. As a transmembrane protein APP can localize in lipid rafts, which are small sterol- and sphingolipid enriched domains that facilitate protein and lipid interactions and play a role in cellular signaling and membrane transport (Hicks et al., 2012). Multiple studies together uncovered that an increase in cholesterol levels promotes APP and BACE1 colocalization in lipid rafts which promotes clathrin-mediated endocytosis and APP processing via the amyloidogenic pathway (Figure 2) (Wahrle et al., 2002; Cordy et al., 2003; Ehehalt et al., 2003; Osenkowski et al., 2008; Cossec et al., 2010; Marquer et al., 2011). This is supported by a recent study in human iPSC-derived neurons, where lowering cholesterol levels reduced the interaction between full-length APP (flAPP) and BACE1, potentially explaining why APP processing is inhibited and flAPP levels are increased upon statin treatment (Langness et al., 2021). In addition, a cholesterol dependent interaction between flotillin and APP in lipid rafts might further promote endocytosis (Chen et al., 2006; Schneider et al., 2008; Cho et al., 2020). Importantly the cholesterol effect is APP specific, as higher membrane cholesterol levels did not affect endocytosis of other membrane proteins like transferrin (Cossec et al., 2010). Also, high membrane cholesterol levels only promote endocytosis and amyloidogenic processing of APP that is localized in lipid rafts, while APP located in the membrane outside of lipid rafts is unaffected (Cho et al., 2020). When analyzing lipid raft composition in postmortem AD vs. control brain tissue, Fabelo et al. (2014) actually detected lower cholesterol, but higher CE presence in lipid rafts of AD subjects, indicating that plasma membrane CE might also contribute to regulation of APP processing. Increased levels of CE, have also been observed both in human postmortem AD brain tissue and in mouse models of AD (Chan et al., 2012; Tajima et al., 2013; Yang et al., 2014).

Cholesterol can also bind directly to APP in the transmembrane C-terminal domain (C99) (Beel et al., 2008; Barrett et al., 2012; Song et al., 2013; Nierzwicki and Czub, 2015). In iPSC derived neurons mutating the cholesterol-binding domain in APP results in reduced APP processing and Aβ production (van der Kant et al., 2019). In addition, inhibition of Aβ secretion by cholesterol-lowering statin treatment depended on the cholesterol binding domain in APP (van der Kant et al., 2019). Export of APP from the ER and subsequent APP processing to Aβ is also cholesterol and/or CE dependent (Puglielli et al., 2001; Hutter-Paier et al., 2004; Huttunen et al., 2009; van der Kant et al., 2019), and regulated by the cholesterol-binding domain in APP (Langness et al., 2021). APP dimerization inhibits exit of flAPP from the ER, and subsequent processing to Aβ (Kaden et al., 2008; Eggert et al., 2009, 2018; Decock et al., 2015; Langness et al., 2021). Interestingly, residues required for binding of APP to cholesterol overlap with residues required for APP dimerization (Song et al., 2013), and thus binding of cholesterol to APP might prevent APP dimerization thereby enhancing ER-export of monomeric APP (Nierzwicki and Czub, 2015; Langness et al., 2021). Whether the cholesterol-binding domain of APP also directly affects APP recruitment into lipid rafts, thereby providing another level of cholesterol-dependent regulation of APP-processing is currently unknown. Also, whether ER cholesterol, or ER CE’s are the main driver of these processes needs to be further established. It is, however, interesting to note, that like SREBP processing, the regulation of APP processing is very much dependent on intracellular cholesterol levels, raising the possibility that APP is a regulator of intracellular cholesterol homeostasis, which is also supported by a number of publications (Box 2). Overall, there is evidently a strong correlation between cholesterol levels and Aβ generation, with increased levels of cholesterol and/or CE in neurons driving Aβ generation (Figure 2).

**Box 2. Tables turned; APP as a regulator of cellular cholesterol metabolism.**

While cholesterol levels are known to regulate APP processing (Figure 2), accumulating data indicate that APP and its cleavage products can also regulate cholesterol metabolism in turn. In primary cultures of rat cortical neurons, higher expression of full length APP decreased HMGCR-mediated cholesterol synthesis, while lowering APP levels increased cholesterol biosynthesis (Pierrot et al., 2013). Similarly, deletion of APP caused increased SREBP2 target gene expression in human iPSC derived astrocytes (Fong et al., 2018). In addition, the C99 APP-fragment has been demonstrated to cluster cholesterol in the ER membrane thereby lowering *de novo* cholesterol synthesis (Montesinos et al., 2020), while the APP intracellular domain (AICD), a cytosolic fragment generated from C99, can directly bind to, and suppress, the LRP1 promoter thereby potentially lowering LRP1 dependent uptake of ApoE delivered cholesterol into neurons (Liu et al., 2007). In line with the position of the cholesterol binding domain in APP (Barrett et al., 2012), Aβ_40_ and Aβ_42_ peptides have been shown to bind extracellular cholesterol, thereby competing with ApoE or LDL driven cholesterol import, and reducing ApoE-dependent cholesterol delivery (Yao and Papadopoulos, 2002). In astrocytes, exogenous Aβ stimulated cholesterol transport from plasma membrane to the Golgi, thereby lowering plasma membrane cholesterol levels (Igbavboa et al., 2009). Finally, Aβ_42_ has been shown to inhibit astrocytic ABCA1 expression (Canepa et al., 2011), which would reduce cholesterol secretion and transport to neurons. Together these results show that APP processing and cleavage fragments can directly affect brain cholesterol homeostasis. This raises the interesting question whether FAD-associated mutations that affect APP-processing also alter brain cholesterol metabolism, which could then further contribute to AD pathology in a cholesterol-dependent manner. Indeed, accumulation of CE has been demonstrated in multiple mouse models of FAD (Chan et al., 2012; Tajima et al., 2013; Yang et al., 2014), indicating that altered brain cholesterol metabolism could hurry pathogenesis also in FAD.

### Cholesterol and Tau

In addition to an established connection between cholesterol and APP processing, cholesterol metabolism was also recently found to directly regulate phosphorylated Tau (pTau) levels in iPSC-derived neurons. As identified by an unbiased high-throughput drug screen, drugs that reduced CE levels in iPSC-derived neurons from familial AD (FAD) patients, also decreased pTau levels (van der Kant et al., 2019). This reduction of pTau was mediated by an increase in proteasomal degradation of pTau, and independent on the effect of CE on APP processing and Aβ (van der Kant et al., 2019). Interestingly, genetically lowering cholesterol esterification in triple-transgenic AD mice (3xTg-AD) mice also lowered pathological Tau accumulation (Shibuya et al., 2015). In addition, *in vivo*, genetic inhibition of ApoE-mediated cholesterol transport from astrocytes to neurons also reduced neuronal pTau levels in mice (Wang et al., 2020). While the exact mechanism underlying cholesterol-dependent regulation of Tau needs to be further established, these findings do further implicate cholesterol as a central player in AD pathogenesis upstream of Aβ and Tau pathology (Kant et al., 2019).

## Dysregulation of Brain Cholesterol in AD; It Is in the Genes

The last decade has seen the discovery of numerous genetic risk factors for LOAD by genome-wide-association studies (GWAS) on LOAD patients vs. healthy controls (Lambert et al., 2013; Jansen et al., 2019; Kunkle et al., 2019; Bellenguez et al., 2020). A high number of the LOAD risk genes have roles in lipid homeostasis, which is best defined for ApoE.

### ApoE

**FIGURE 3:**
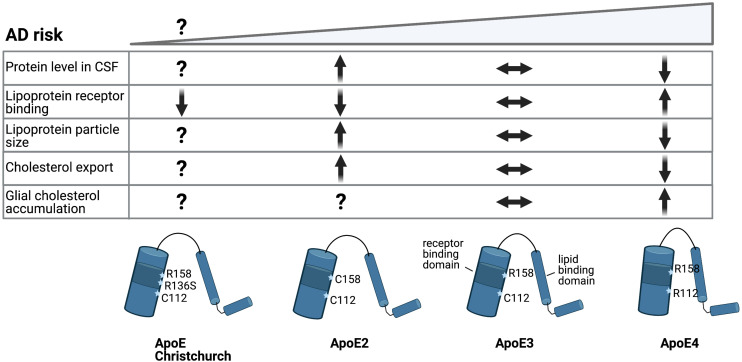
Alzheimer’s disease risk variants in ApoE affect cholesterol metabolism. Schematic representation of ApoE allelic variants and their consequences. Single amino acid changes in ApoE confer different risk for LOAD. Conformational change of the protein structure by expression of the different ApoE alleles changes LDL receptor binding and lipid particle binding capacity, which might affect brain cholesterol metabolism (BioRender, 2021).

Three common allelic ApoE genetic variants exist in the human population; ApoE2, ApoE3 and ApoE4. ApoE3 is the most common isoform present homozygous in over 60% of the population and is considered the reference allele for LOAD risk (Jeong et al., 2019). ApoE4 is a strong risk factor for LOAD: carriers of one ApoE4 allele have a 3 to 4-fold increased risk for LOAD, while homozygous ApoE4 carriers have an approximate 14-fold increased risk of developing LOAD compared to ApoE3 carriers (Liu et al., 2013). It has to be noted, however, that the penetrance of the ApoE4-risk allele varies in different ethnicities, possibly due to differences in ApoE expression levels (Griswold et al., 2021). In contrast to ApoE4, expression of the ApoE2 allele confers a decreased risk for LOAD and hence is considered protective (Corder et al., 1994; Reiman et al., 2020). Despite their strong effects on LOAD risk, the three ApoE isoforms only differ from each other by two amino acids (Chen et al., 2020). ApoE3 contains a Cys112 and Arg158, of which Cys112 is changed to Arg112 in ApoE4 and Arg158 is changed to Cys158 in the ApoE2 variant (Figure 3). As an apolipoprotein ApoE interacts with lipoproteins to execute its function as a cholesterol and lipid carrier. Via a receptor binding domain ApoE can interact with lipoprotein receptors to be internalized and deliver the cargo of lipids to cells. As described above, this ApoE-dependent route is crucial for transport of cholesterol and cholesterol precursors from astrocytes to neurons in the mature brain.

Strikingly enough, while peripheral ApoE4 is a major risk factor for hypercholesterolemia (Box 1), it remains largely unknown how ApoE isoforms affect cholesterol metabolism in the brain. Astrocytes are the highest ApoE-expressing cell type in the brain (Huang et al., 2004), and transcriptomic analysis of human iPSC-derived neurons, astrocytes and microglia, revealed that ApoE4 driven changes in gene expression were most dramatic in astrocytes (Julia et al., 2019). Compared to ApoE3 astrocytes, ApoE4 astrocytes expressed higher levels of genes with a role in cholesterol biosynthesis and displayed cholesterol accumulation in lysosomes, while CE levels were not increased (Lin et al., 2018; Julia et al., 2019). The dysregulation in lipid metabolic genes in ApoE4 astrocytes was also confirmed in human control and AD brain samples (Julia et al., 2019). Also, an increased number of smaller lipid droplets has been detected in ApoE4 astrocytes compared to ApoE3 astrocytes derived from human ApoE-replacement mice (Farmer et al., 2019).

How ApoE4 affects cholesterol metabolism in other brain cell types like neurons and microglia is even less clear. ApoE4 expressing human iPSC-derived astrocytes showed reduced support of neuronal survival in an iPSC-derived neuron-astrocyte co-culture compared to ApoE3 expressing astrocytes (Zhao et al., 2017). One way by which different ApoE polymorphisms could affect total brain lipid metabolism is by altering the export of ApoE-lipoprotein particles (Figure 3). In human CSF and upon overexpression in mice, ApoE2 has been shown to generate bigger HDL particles compared to ApoE3, while ApoE3 in turn is associated with bigger HDL particles then ApoE4, suggesting less sterol transport by ApoE4 (Hu et al., 2015; Heinsinger et al., 2016). Accordingly, an isoform dependent effect on cholesterol efflux, ApoE2 > ApoE3 > ApoE4, was detected previously in primary rat or mouse astrocytes and neurons (Michikawa et al., 2000; Rawat et al., 2019). No isoform dependent changes in binding of ApoE to ABCA1 were found that could explain reduced cholesterol efflux from ApoE4 expressing astrocytes, although ApoE4 has been suggested to affect ABCA1 membrane trafficking (Krimbou et al., 2004; Rawat et al., 2019). In addition, ABCA1 has recently been identified as a LOAD risk gene itself further implicating this pathway in LOAD pathogenesis (Bellenguez et al., 2020). In addition to export, ApoE genotype might also differentially affect the internalization of ApoE-lipoprotein particles. Lipidation of the ApoE protein triggers a conformational change that increases its binding affinity for the LDL receptors. Lipidated ApoE4 shows the strongest binding affinity for LDLR, while the binding of lipidated ApoE2 to LDLR is reduced compared to ApoE3 (Figure 3) (Chen et al., 2020). How these differences in receptor binding affinity affect uptake of ApoE-lipoprotein particles in specific brain cell types remains to be determined. The protein levels of ApoE also differ between isoforms, where ApoE2 levels are highest and ApoE4 levels are lowest in CSF and plasma (Castellano et al., 2011; Cruchaga et al., 2012). This might be a result of their different receptor affinities, as LDLR loss caused an increase in ApoE3 and ApoE4 levels, but not ApoE2 levels (Fryer et al., 2005). Importantly, the recently described Christchurch mutation in *APOE* (R136S) results in strongly reduced LDLR binding of ApoE, similar to ApoE2 (Figure 3). Presence of this mutation has been reported in an individual who had no signs of cognitive decline or Tau-pathology until advanced age despite carrying a PSEN1 mutation that causes autosomal-dominant AD (Arboleda-Velasquez et al., 2019). These results suggest a strong protective effect of the Christchurch ApoE variant, although this conclusion awaits further confirmation. Together, these results suggest strong ApoE genotype dependent effects on cholesterol metabolism in astrocytes. Whether, and how, this affects the proper astrocyte-dependent support of neuronal function and/or downstream AD pathology in neurons and glia remains to be determined.

Besides ApoE, other AD risk genes also have a predicted role in lipid metabolism. This is established for AD-genes that act in similar processes as ApoE and ABCA1, such as CLU and ABCA7 (discussed below), but also increasingly recognized for genes which are highly, or exclusively, expressed in microglia (discussed in the next section).

### CLU and ABCA7

Like ApoE, clusterin (CLU) is a component of lipoproteins. The *CLU* gene producing clusterin (also referred to as ApoJ) is expressed in astrocytes and has a wide range of biological functions including cholesterol and lipid transport. In periphery, clusterin can form HDL particles that are transported to the liver (Baralla et al., 2015; Foster et al., 2019). Also, clusterin is expressed upon damage in arteries and could remove cholesterol from macrophage-foam cells that promote formation of atherosclerotic lesions (Gelissen et al., 1998; Foster et al., 2019). Clusterin plasma levels have been shown to correspond to severity of AD in patients (Thambisetty et al., 2010; Jongbloed et al., 2015), yet the specific role in brain cholesterol homeostasis is unknown.

Another AD risk gene, ABCA7 shares 54% homology with ABCA1, the protein known to load cholesterol onto ApoE particles (Kaminski et al., 2000). However, what role ABCA7 has in intracellular cholesterol- and lipid transport remains unclear. ABCA7 is highly expressed in the brain, predominantly in neurons and microglia (Zhang et al., 2014). Reduced ABCA7 levels are observed in AD brain (Lyssenko and Praticò, 2020) and hippocampus of mice on a high fat diet (Zou et al., 2020). Contrary to ABCA1, transcription of ABCA7 is downregulated when cholesterol levels are high in the cell (Iwamoto et al., 2006). Recent studies on detergent purified ABCA7 showed that removal of cholesterol led to increased ATPase activity in ABCA7 (Le et al., 2021). In addition, ATPase activity was stimulated by interaction with apolipoproteins, ApoA-I and ApoE, where ATPase activity was hardly stimulated in presence of the ApoE4 compared to the ApoE3 variant. Interestingly, also the ApoE2 variant had a lower effect in stimulating ATPase activity of ABCA7 compared to ApoE3 (Le et al., 2021). The physiological consequences of these ApoE genotype dependent effects on ABCA7 and cholesterol metabolism remain to be determined. *In vitro* analysis in baby hamster kidney (BHK) cells transfected with either ABCA1-GFP or ABCA7-GFP revealed that free cholesterol efflux through ABCA7 was much lower compared to ABCA1 and was unaffected by ApoE genotype, while cholesterol efflux by ABCA1 was greatly reduced in presence of ApoE4 (Tomioka et al., 2017). Interestingly, recent data on the ABCA7 homolog in Drosophila suggests that ABCA7 might play a role in the neuron-to-glial transport of lipids to protect neuronal functionality and viability from toxic accumulation of peroxidized lipids, for instance generated by oxidative stress (Moulton et al., 2021). These results open the door for future studies on the role of ABCA7 in brain cholesterol metabolism and AD development.

## Cholesterol as a Driver of (Micro)Glial Dysfunction and Gliosis

### TREM2, PLCγ2, and Microglial ApoE

Multiple LOAD risk genes such as ApoE, TREM2 and PLCγ2 are highly -or exclusively- expressed in (micro)glia and have been shown to regulate lipid metabolism and microgliosis. Increased expression of genes associated with lipid metabolism are found in microglia during development, damage or disease (Efthymiou and Goate, 2017; Keren-Shaul et al., 2017; Hammond et al., 2019; McQuade and Blurton-Jones, 2019). A good example is triggering receptor expressed on myeloid cells 2 (TREM2), which in brain is primarily expressed in microglia. Carriers of the R47H or R62H variant in this gene have an up to 4-fold increased risk for LOAD (Ulrich and Holtzman, 2016). TREM2 has multiple ligands including the apolipoproteins ApoE and clusterin, and binding of TREM2 to these proteins is enhanced by their lipidation (Atagi et al., 2015; Bailey et al., 2015; Yeh et al., 2016). Binding of TREM2 to lipoprotein particles is reduced by the TREM2 R47H variant, which could lead to altered cholesterol load in microglia and also affects phagocytosis of lipoprotein bound Aβ by microglia (Yeh et al., 2016). Both astrocytes and microglia can phagocytose Aβ, thereby contributing to Aβ clearance from the brain (Tarasoff-Conway et al., 2015; Ries and Sastre, 2016). Single-cell RNA-sequence (RNA-seq) data from AD mouse models revealed so-called disease associated microglia (DAM) as a reactive microglial population that is generated when AD pathology is present in the brain (Keren-Shaul et al., 2017). The transition to a DAM-phenotype was dependent on TREM2 (Zhou et al., 2020). DAM have reduced expression of several homeostatic microglial genes accompanied by a significant increase in expression of lipid metabolism and phagocytosis genes, including ApoE. These activated DAM microglia localize predominantly around amyloid plaques where they form a neuroprotective barrier, prevent propagation of Tau pathology in mice and are believed to play a role in Aβ clearance (Condello et al., 2015; Yuan et al., 2016; Keren-Shaul et al., 2017; Leyns et al., 2019). Of note, although increased ApoE levels were detected by recent (single-cell and single-nuclear) RNAseq studies in microglia from human AD postmortem brain tissue, a subpopulation with DAM signature was not identified (Grubman et al., 2019; Olah et al., 2020; Srinivasan et al., 2020; Zhou et al., 2020). While this may indicate differences between AD pathogenesis in mouse models and in humans, it could also be a consequence of technical limitations, e.g., too few single microglial cells analyzed or the low sensitivity of single-nuclear RNAseq to detect microglial activation genes (Del-Aguila et al., 2019; Mathys et al., 2019; Thrupp et al., 2020). As a consequence of TREM2 loss, microglia from *TREM2^–/–^* mice on a demyelinating cuprizone (CPZ) diet failed to upregulate DAM-genes needed for cholesterol transport and lipid metabolism like ApoE, and could not clear myelin derived cholesterol, leading to microglial CE accumulation. A similar accumulation of CE was observed in human TREM2 knock out iPSC-derived microglia-like cells (iMG), when these were treated with exogenous myelin (Nugent et al., 2020). The role of TREM2 in lipid metabolism is (at least in part) mediated by the enzyme phospholipase C γ2 (PLCγ2), for which a LOAD-protective variant has been identified (P522R). PLCγ2 is an intracellular enzyme, which is also specifically expressed in microglia. The protective P522R variant is associated with a gain of function and hence loss of TREM2 or PLCγ2 are both expected to negatively affect LOAD risk (Magno et al., 2019). Indeed, TREM2 or PLCy2 deficient iMG showed a similar defect in upregulation of genes needed for lipid metabolism. Accordingly, both TREM2- and PLCy2-deficient iPSC derived microglia fail to clear cholesterol after phagocytosis of myelin debris (Andreone et al., 2020; Nugent et al., 2020). Analysis of the lipidome after myelin treatment revealed accumulation of free cholesterol, CE, myelin-derived ceramides [Cer, hexosylceramides (HexCer), sulfatides and diacylglycerols (DAGs) and triacylglycerols (TAGs) in the TREM2- and PLCy2-deficient iMG compared to WT iMG (Andreone et al., 2020)]. Importantly, Andreone et al. (2020) show that expression of the LOAD-protective variant P522R reduced CE accumulation to a greater extent than WT PLCy2, further indicating that TREM2 and PLCy2 work together in microglial lipid metabolism and demonstrating the relevance of this pathway for LOAD development.

Accumulation of CE was also observed in sorted microglia from *ApoE^–/–^* mice, indicating that ApoE dependent transport prevents cholesterol overload in microglia (Nugent et al., 2020). ApoE levels are increased in AD-brain microglia and both intracellular and extracellular clearance of Aβ is greatly facilitated by lipidated ApoE particles (Jiang et al., 2008). Lipidated ApoE can directly interact with Aβ to support phagocytosis. In addition, independent of a direct ApoE-Aβ interaction, depletion of cellular cholesterol from microglia via ApoE-containing HDL particles promoted Aβ degradation in primary mouse microglial cultures (Lee et al., 2012). How the TREM2-PLCγ2-ApoE axis contributes to LOAD development remains an important question for future studies. Whether the effects of the LOAD risk genes on AD pathology are directly coupled to the function of these genes in (micro)glial cholesterol metabolism is currently unknown, but of great interest to the field.

### From Cholesterol Dysregulation to (Micro)Gliosis

Glial LOAD risk genes that affect (micro)glial cholesterol metabolism could impact AD pathology via their effect on gliosis, represented by accumulation of reactive astrocytes and immune activated microglia (Shi and Holtzman, 2018). Gliosis is detected early in AD development, and seen as a major pathological hallmark (Heneka et al., 2005; Carter et al., 2012). While considered primary a neuroprotective response, gliosis is also thought to contribute to progressive AD development (Heneka et al., 2015). Changes in cholesterol metabolism have been linked to gliosis by various studies. For example, a high cholesterol diet induces astrocytic activation and increased expression of ApoE in mice (Chen et al., 2016). In line, exogenous cholesterol addition in rat astrocytes triggered astrocyte activation, indicated by upregulation of glial fibrillary acidic protein (GFAP) (Avila-Muñoz and Arias, 2015). Finally, exogenous addition of a mixture of oxysterols, representing oxysterols that are produced when cholesterol accumulates in the AD brain, promote upregulation of reactive astrocyte markers, which contributed to synaptotoxicity (Staurenghi et al., 2021). In microglia, high cholesterol can affect immune function, as particularly studied in respect of cholesterol-rich myelin debris, which promotes inflammatory activation of microglia (Cantuti-Castelvetri et al., 2018). Myelin debris treatment in bone-marrow derived macrophages from mice caused NLRP3 inflammasome activation, possibly due to lysosomal rupture after formation of cholesterol crystals (Cantuti-Castelvetri et al., 2018). Mechanistic understanding of altered immune activation in microglia upon high cholesterol load requires further studying. In macrophages, changes in membrane cholesterol load have also been shown to affect lipid raft composition and TLR mediated signaling, where high cholesterol levels cause hyperresponsiveness to LPS treatment (Fessler and Parks, 2011). In addition, high cholesterol levels could drive CD36-dependend inflammatory signaling via inhibition of Nrf1 in the ER (Widenmaier et al., 2017). As described above reversed cholesterol transport is needed to revert the pro-inflammatory state in microglia and promote re-myelination (Cantuti-Castelvetri et al., 2018). Microglia increase post-squalene sterol synthesis in response to cholesterol overload to activate LXR dependent transcription and promote cholesterol export (Berghoff et al., 2021). Increased secretion of pro-inflammatory cytokines IL-1β and IL-18 upon myelin treatment combined with TLR activation in TREM2 KO iMG suggests that increased sterol levels might indeed contribute to excessive inflammatory responses by microglia, which might be further enhanced in presence of LOAD risk genes (Andreone et al., 2020). Moreover, Lin et al. (2018) recently suggested that ApoE4 expression in microglia might be sufficient to convert them into an immune-active state. In line, ApoE4 expressing primary mouse microglia respond stronger to immune activation compared to ApoE2- or ApoE3 expressing microglia (Wong et al., 2020). Whether these processes are mediated by altered cholesterol metabolism in glia downstream of ApoE remains to be established.

## Conclusion

Cholesterol is a central player in AD affecting Amyloid, Tau and gliosis. In addition, LOAD genetic risk factors point to a strong effect of lipid metabolism in AD development. Yet, mechanistic understanding of the pathways by which dysregulation of cholesterol metabolism contributes to AD development remains largely lacking. A number of major outstanding questions are; (1). How is cholesterol metabolism affected in each specific brain cell type in AD patients (2). How do LOAD-risk genes affect cholesterol metabolism in specific brain cell types and the transport of lipids between these cells (3). How do changes in brain cholesterol metabolism contribute to AD pathology (Amyloid, Tau, and gliosis) and finally (4) is cholesterol itself or one of its derivatives most toxic in this context?

**FIGURE 4:**
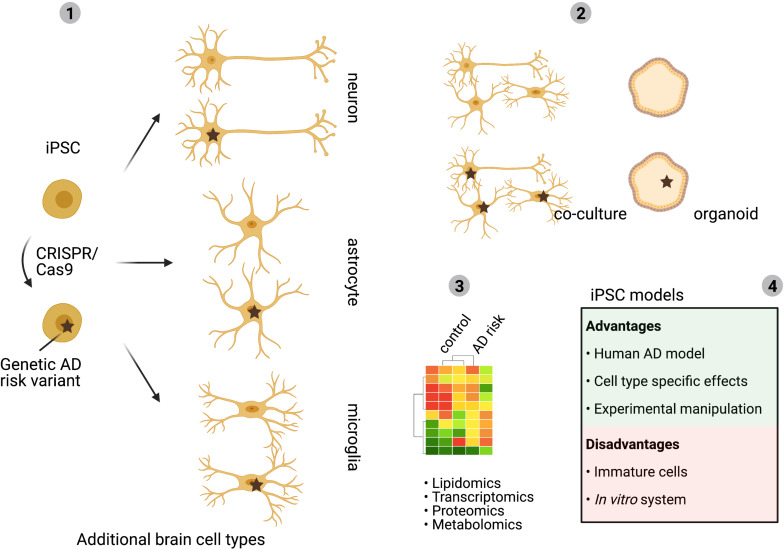
Human iPSC models to study cell type specific effects. Multiple LOAD-risk variants are found in genes with an expected role in lipid metabolism and changes in cholesterol metabolism are linked to AD development. Yet, how individual LOAD-risk variants affect brain cholesterol metabolism and AD pathology is largely unclear. As described in this review, the complex organization of brain cholesterol metabolism depends on cell intrinsic metabolism as well as on the transport of cholesterol between the different brain cell types. Development of human iPSC derived cell models allows for separation of these processes and gives the possibility to introduce risk variants in each selected brain cell type (1). For example, analysis of cell type specific effects of LOAD-risk gene expression will uncover in which cell type a LOAD-risk variant has the biggest impact. Co-culture models of different brain cell types can subsequently be used to identify how LOAD-risk variants affect lipid transport, cholesterol metabolism between different brain cell types and how this affects downstream AD pathology (2). iPSC-derived brain cell models allow for -omics approaches to determine complex cell type dependent effects (3). Advantages and disadvantages of iPSC-models are discussed in (4). Mechanistic insights acquired by iPSC studies can contribute to the identification of novel (cell type) specific targets for future therapy development (BioRender, 2021).

Progress in technology development has delivered new tools to address these questions in human cells and tissue. Techniques that will help to answer the outstanding questions include the generation of human brain cell types from induced pluripotent stem cells and introduction of specific LOAD-risk mutations by CRISPR/Cas9 gene-editing (Figure 4). This approach allows the mapping of cell type specific effects of LOAD-risk variants on cholesterol metabolism combined with the possibility to mechanistically study AD pathology for a certain genetic background. Extension of this approach to co-culture or 3D organoid models of different iPSC derived brain cell types gives the opportunity to further study complex interplay between different cell types of the brain (Figure 4). Finally, single-nuclear- or single-cell-RNA sequencing of postmortem brain tissue, or 3D iPSC derived brain cell models, with distinct LOAD-risk genotypes will result in comprehensive data on cell type specific effects on gene expression. In addition, subcellular populations that might impact cholesterol metabolism or AD pathology can be identified by this approach. A better mechanistic understanding of the cholesterol-dependent pathways that drive (early) AD development will also uncover novel (cell type) specific targets for rational drug discovery. An example of such a drug discovery effort for brain-lipid targeting drugs is the discovery that Efavirenz, an FDA approved HIV-drug, activates the neuronal specific enzyme CYP46A1 to promote conversion of excess cholesterol to 24S-OHC that can be secreted from the brain via the BBB. Efavirenz lowered pTau levels in iPSC-derived neurons from AD patients and improved behavior in 5xFAD mice (Petrov and Pikuleva, 2019; Petrov et al., 2019; van der Kant et al., 2019). A phase I clinical trial with intermediate-to-high doses of Efavirenz has started in patients with MCI in the United States (Nugent et al., 2020) and a similar trial with low-dose Efavirenz is planned to start in the Netherlands.

The approaches described above, and the rapidly increasing knowledge on brain lipid metabolism, will contribute to tackling the outstanding questions in this field and will undoubtedly provide much needed new insights on AD etiology and the role of cholesterol metabolism in this process. Such knowledge will likely be fundamental to develop targeted therapies to prevent, delay or cure AD in the future.

## Author Contributions

FF and RK provided the original conception and design of the manuscript and revised the manuscript. FF researched data and wrote the manuscript to which RK provided feedback. Both authors contributed to the article and approved the submitted version.

## Conflict of Interest

The authors declare that the research was conducted in the absence of any commercial or financial relationships that could be construed as a potential conflict of interest.
